# Wholegrain Durum Wheat Bread Fortified With Citrus Fibers: Evaluation of Quality Parameters During Long Storage

**DOI:** 10.3389/fnut.2019.00013

**Published:** 2019-02-13

**Authors:** Alfio Spina, Selina Brighina, Serena Muccilli, Agata Mazzaglia, Simona Fabroni, Biagio Fallico, Paolo Rapisarda, Elena Arena

**Affiliations:** ^1^Centro di Ricerca Cerealicoltura e Colture Industriali, Consiglio per la Ricerca in Agricoltura e l'Analisi dell'Economia Agraria (CREA), Acireale, Italy; ^2^Dipartimento di Agricoltura, Alimentazione, Ambiente (Di3A), University of Catania, Catania, Italy; ^3^Centro di Ricerca Olivicoltura, Frutticoltura e Agrumicoltura, Consiglio per la Ricerca in Agricoltura e l'Analisi dell'Economia Agraria (CREA), Acireale, Italy

**Keywords:** durum wheat bread, citrus fibers, storage, microbiological assay, HMF, sensory attributes

## Abstract

The aim of this work was to evaluate the effect of the addition of citrus fibers, from blood orange and lemon peels to produce a functional durum wheat bread. Breads fortified in fiber were packaged under a modified atmosphere (MAP) and stored at 25°C up to 120 days. No significant differences were observed with respect to the specific volume and weight, internal structure, pH and titratable acidity among the bread samples obtained using different types and percentages of fibers. Storage time, at 30 up to 90 days, affected significantly the bread firmness and caused significant differences in 5-hydroxymethylfurfural (HMF) levels in all bread samples. In fortified breads with citrus fibers the yeast and mold counts showed values of approximately 1 log10 cfu/g for the first 30 days and 3.5 log10 cfu/g at the end of storage. The results of the sensory evaluation highlight that loaves enriched with blood orange and lemon fibers showed a citrus flavor but had a similar overall evaluation respect to control bread produced without addition of citrus fiber. The results of this study showed that the addition up to 2% of blood orange and lemon fibers in wheat whole durum flour is a possible strategy to produce “high fibre” bread.

## Introduction

Dietary fiber have an important role in maintaining good health and prevention of disease. The increase of the dietary fiber intakes was associated with the reduction of cardiovascular disease and the incidence of type 2 diabetes (1–4) and with the prevention of excessive weight gain, thanks to the role played on the regulation of appetite (5, 6).

The majority of the world's population consumes bread daily and it represents the ideal food to act as a vehicle for healthy substances. The addition of dietary fiber, generally modified the physicochemical characteristics both of dough and bread (7). Several authors reported the successful use of fruit and vegetable fiber in bakery products (8–11). In southern Italy, particularly in Sicily, citrus industry produces approximately 500,000 t/year of “pastazzo,” a by-product derived from the industrial squeezing of citrus fruit, which currently presents serious disposal problems. After numerous washings and purifications, it is possible to obtain citrus flour rich in dietary fiber, which can be added to food products.

Several authors used citrus fiber to fortify baked goods. A decrease of bread volume without significant worsening of the crumb texture and various detrimental effects on dough handling and bread quality with the replacement of flour with dietary fiber was reported (12, 13). Nassar et al. (14), suggested that 15% orange peel and pulp could be incorporated in biscuits, as a source of dietary fiber containing bioactive compounds (flavonoids, carotenoids, etc.). Biscuits of good technological quality, good level of acceptance and a decreased energy value, were obtained by replacing up to 15 g/100 g of the wheat flour with extruded orange pulp (15). A reduction both of energy content and *in vitro* protein digestibility was reported for cookies added with apple or lemon or wheat bran fiber (16); the partial replacement of wheat flour with a dietary fiber-rich orange bagasse allowed to produce baked products containing high levels of total dietary fiber and indigestible fraction and a decrease of glycemic index of the products (17). During baking process, the heat transmission favors, mainly in the bread crust, the formation of Maillard reaction products, that improve the flavor, color and texture of food products (18). 5-Hydroxymethylfurfural (5-HMF) is a product both of the Maillard reaction and of the acid-catalyzed thermal dehydration of hexose. In several processed foods is used as index of thermal abuse (19–21) and in baking products HMF levels make possible to monitor the heating processes (22, 23). Estimated dietary intakes range between 4 and 350 mg per person and day, and among foods, cereal and cereal products, in particular bread, may serve as a major source of HMF exposure. In cereal products the average concentrations of HMF ranged from 14 to 53 mg/kg (24, 25) and the average intake can vary greatly with regard to low or high consumption. Even if, seems, that the toxic potential of HMF is rather low several strategies were used to reduce the HMF level in bakery products (26–28).

The aim of this work was to evaluate the effect of the addition of different level of citrus fiber on the physicochemical, microbiological, and sensory properties of durum wheat whole bread. Moreover, the evolution both of the quality parameters and the sensory attributes were studied during a long storage time under MAP conditions.

## Materials and Methods

### Materials

Durum wheat whole semolina was provided by the “Valle del Dittaino” Agricultural Cooperative Society a.r.l. (Assoro, Enna, Italy), a local industrial bakery. The bread ingredients were food grade. The citrus fibers were derived from blood oranges (*C. sinensis* L. Osbeck) and lemons (*C. limon* L. Burm f.) and were kindly donated by Ortogel S.p.A., Caltagirone, Italy. The compressed yeast (Mauri Extra Classic) was obtained from “AB Mauri Italy S.p.A.,” Pavia, Italy and the NaCl was from “Mulino S. Giuseppe,” Catenanuova, Italy.

### Methods

#### Physicochemical Analysis of Durum Wheat Whole Flour

Ash content was obtained following the ISO method 2171 (2007). The gluten index was determined using a Glutomatic 2200 apparatus (Perten Instruments AB, Huddinge, Sweden) according to the method UNI 10690 (1979). The α-amylase activity was determined using the Falling Number 1500 apparatus (Perten Instruments AB, Huddinge, Sweden), following the method UNI EN ISO 3093 (2010).

Total, soluble and insoluble dietary fiber content was determined using a K-TDFR kit from Megazyme (Megazyme International, Bray, Ireland) (29). The protein content and color indexes were determined according to the methods described for durum wheat whole flour (30).

Rheological dough properties were evaluated by mixograph (National Mfg. Co, Nebraska, USA) and farinograph curves (Brabender instrument, Duisburg, Germany) (31). All the analyses were conducted in triplicate.

#### Chemical Characterization of Citrus Fiber

Total, soluble and insoluble dietary fiber content was determined as already reported above (29). The moisture content was determined according to the ISO method 712:2009. The ash content was obtained according to the ISO method 2171:2007. The alkalinity of the total ash was carried out following ISO method 5520:1981 and was expressed as the alkalinity index (the number of milliliters of 1 N acid solution required to neutralize 1 g of the ash obtained from the sample). The protein amount was determined by the Kjeldhal method. The sugars were determined by using HPLC with a refractive index detector (32). The pectin content was titrimetrically quantified as galacturonic acid according to the legal pectin specification (33). The water binding capacity (WBC), the water tendency to associate with hydrophilic substances, was determined (34). All the analyses were conducted in triplicate.

#### Bread-Making Process and Storage Conditions

The breads were produced in an industrial bakery (“Valle del Dittaino”—Agricultural Cooperative Society a.r.l., Assoro, Italy). For each dough, 68 kg of durum wheat whole flour was mixed with tap water (78 ± 3%), compressed yeast (0.5%), NaCl (2.6%) and blood orange (OF) and/or lemon fibers (LF). In the control dough no citrus fiber were added; whereas in the other four doughs citrus fiber were added in variable amount: 1.5 and 2.0% of orange fiber (code samples 1.5OF and 2.0OF, respectively) or lemon fiber (code samples 1.5LF and 2.0LF, respectively) or a mix of orange and lemon fiber (1.0 + 1.0%) (code samples 1.0 + 1.0OLF). These levels were chosen to obtain breads with a total dietary fiber content higher than 6 g/100 g, which is the minimum required limit to indicate that a food is “high in fibre” (35). All the ingredients were mixed for 13 min using a mixer with beater arms (IBT 300, Pietroberto S.p.A, Marano Vicentino, Italy). The tap water temperature was 22°C, and at the end of mixing, the dough temperature was 25.9 ± 0.4°C. All the other ingredients were expressed as a % of the weight of whole wheat durum flour.

Afterward, the dough was broken into approximately 1,150 g pieces using volumetric dividing (Omega, Pietroberto S.p.A, Marano Vicentino, Italy), obtaining approximately 100 loaves for each dough. The loaves were rounded in a conical rounder (CO 3000, 1500 pieces/h, Turri F.lli S.r.l., Costa di Rovigo, Italy) and leavened in a tray proofing cell (Pavailler Engineering S.r.l., Galliate, Italy) for 2 h and 45 min at 32°C with a relative humidity (RH) of 65%.

The loaves were baked for 1 h at 210°C in a continuous oven (Pavailler Engineering S.r.l., Galliate, Italy). After 2 h of cooling in a conditioned room at 20°C with a RH of 60%, the breads were packaged under a modified atmosphere in T6011B film (275 μm thickness) (Sealed Air, Cryovac, Italy), and T9250B film (125 μm thickness) (Sealed Air, Cryovac, Italy) was used to cover and seal the package. The containers were vacuumed, filled with mixed gas and sealed with a Multivac R-230 packaging machine (Germany) equipped with a modified atmosphere (MAP) mix 9,000 gas-mixer (PBI–Dansensor A/S, Ringsted, Denmark). The trays were packed using 70% N_2_:30 % CO_2_ gas combinations. All packaged bread samples were stored at 25 ± 2°C and 60 ± 2% RH for 4 months and were periodically (0, 30, 60, 90, and 120 days of storage) sampled. Nine loaves for each formulation were withdrawn each month and immediately analyzed. The CO_2_ and O_2_ concentrations in the samples were measured using Check Point (Dansensor PBI, Ringsted, Denmark) gas analyzing equipment.

The following properties were tested for each bread sample during each sampling: volume, height, weight, diameter, crumb porosity, internal structure, loaf firmness, crust thickness, crust and crumb color, moisture, pH, acidity, HMF content, microbiological, and sensory analysis. All the analyses were conducted in triplicate.

### Bread Quality Evaluation

#### Determination of the Physicochemical Properties of Breads

The physicochemical properties of bread samples were evaluated according to Spina et al. (31).

The volume was determined according to the rapeseed displacement in a loaf volume meter, and the specific volume (cubic meters per gram) was calculated as a ratio of the loaf volume and the bread weight. The internal structure was visually estimated, and the crumb porosity was estimated using the Mohs scale. Loaf crust thickness was measured using a digital caliper (Digi-MaxTM, Scienceware^®^, NJ, U.S.A.), on the loaf basis of three central slices, after removing the crumb. The loaf firmness was measured using a texture analyser (Zwick Z 0.5 Roëll, Germany) equipped with an aluminum 8-mm- diameter cylindrical probe. The resulting peak force was measured in kilograms per cubic centimeter.

The CIE L^*^a^*^b^*^ color parameters were measured for the crumb, in the transversely cut bread and on the crust surface, averaging ten distinct points in each case, using a Chroma Meter (CR-200, Konica Minolta, Osaka, Japan) with illuminant D65.

The moisture content was determined in triplicate by gravimetric analysis. The bread samples were ground in a home grinder (La Moulinette, Moulinex, 2002), and then portions of the ground bread sample were placed in an oven at 105°C until the dry weight was constant.

The pH and the total titratable acidity (TTA) were measured in triplicate, using a pHmeter (Mettler Toledo, MP 220). The TTA results were expressed, for the dry matter, as milliliters of 0.1 M NaOH consumed (36).

Total, soluble and insoluble dietary fiber content was measured in 1 g of dried sample using a K-TDFR kit from Megazyme (Megazyme International, Bray, Ireland) (29).

#### HMF Extraction and HPLC Analysis

The HMF was determined according to Spina et al. (31). The bread samples were ground in a home grinder (La Moulinette, Moulinex, 2002); subsequently, an aliquot of the ground sample (5 g) was transferred into a volumetric flask (50 ml), and 25 ml of water was added (JT. Baker, Deventer, Holland). The solution was stirred for 10 min, and then the sample was diluted up to 50 ml with water (JT. Baker, Deventer, Holland) and centrifuged for 45 min at 5,000 rpm. An aliquot of the supernatant was filtered through a 0.45-μm filter (Albet) and injected into an HPLC system (Shimadzu Class VP LC-10ADvp) equipped with a DAD (Shimadzu SPD-M10Avp). The column was a Gemini NX C18 (150 × 4.6 mm, 5 μm) (Phenomenex), fitted with a guard cartridge packed with the same stationary phase. The HPLC conditions were the following: isocratic mobile phase, 90% water at 1% of acetic acid and 10% methanol; flow rate, 0.7 ml/min; injection volume, 20 μl (20). All of the solvents used were HPLC purity grade: water from J.T. Baker, and acetic acid and methanol from Merck. The wavelength range was 220–660 nm, and the chromatograms were monitored at 283 nm.

HMF was identified by splitting the peak of the HMF from the bread-solution sample with a standard of HMF (*P* > 98% Sigma-Aldrich, St. Louis, Mo., U.S.A.) and by comparison of the UV spectra of the HMF standard with that of the bread samples. All analyses were performed in duplicate, including the extraction procedure, and the reported HMF concentration is therefore the average of four values. The results were expressed as mg of HMF per kilogram of the bread dry matter.

#### Microbiological Analysis

The microbiological analysis of the bread samples was performed in triplicate at 0, 30, 60, 90, and 120 days of storage, according to Spina et al. (31). Ten grams of each loaf were aseptically weighed and homogenized using a Stomacher (Brinkmann, Westbury, NY, USA) for 5 min and were serially diluted in a sterile physiological solution (0.9% NaCl). Serial dilutions of the suspension were pour-plated in duplicate onto the following media: plate count agar (PCA) (Oxoid) for total viable count; violet red bile glucose agar (VRBGA) (Oxoid) for Enterobacteriaceae; and Saboraud dextrose agar for yeasts and molds. The VRBGA plates were incubated at 32°C for 48 h under aerobic conditions. The PCA plates were incubated at 30°C for 4 days, and the yeast-count plates were incubated at 25°C for 4 days.

### Sensory Evaluation

The sensory profile was determined by applying the UNI 10957 (2003) method and was defined by a panel of 12 judges (six females and six males) recruited among student and University staff that have chosen to participate to the research and signed the informed consent as our institution do not have an ethics committee for the taste and food quality evaluation studies. The judges were submitted to training over 4 weeks to generate attributes using handmade and industrial breads (31, 37, 38) and to familiarize themselves with the scales and procedures (UNI EN ISO 8586, 2012). The judges, using a scale between 1 (absence of the sensation) and 9 (extremely intense), evaluated the intensity of the eighteen sensory attributes selected on the basis of frequency (%) (39) (Table 1). The evaluation sessions, which were performed at 0, 30, 60, 90, 120 days of storage, were conducted in an equipped laboratory (UNI EN ISO 8589, 2010) from 11:00 to 12:00 a.m. in individual booths illuminated with white light. All data were acquired by a direct computerized registration system (FIZZ Biosystemes, ver. 2.00 M, Couternon, France). The data reported were expressed as the mean ± standard deviation.

**Table 1:** Descriptive terms used for sensory profiling of bread.

	**Attributes**	**Definition**	**Scale anchors**	
Crumb appearance	Crumb color	Color intensity of crumb	Whitish	Light yellow
	Alveolar structure	Porosity of crumb	Fine and uniform	Coarse and poorly homogeneous
Visual-tactile	Humidity	Humidity perceived at the surface of bread crumb	Dry	Humid
Aroma/Flavor	Bread	The typical aroma/flavor of bread just taken out of the oven	Weak	Strong
	Yeasty	The aroma/flavor of a fermented yeast-like	None	Strong
	Citrus	The typical aroma/flavor of citrus	None	Strong
	Off-odor/ Off-flavor	Aroma/Flavor unpleasant, not characteristic of bread perceived through taste and smell when swallowing	None	Strong
Taste	Sweet	A basic taste factor produced by sugars	None	Strong
	Salty	A basic taste factor produced by sodium chloride	None	Strong
	Sour	A basic taste factor produced by acids	None	Strong
	Bitter	A basic taste factor produced by caffeine	None	Strong
Mouthfeel	Astringent	Sensory perception in the oral cavity that may include drying sensation and roughing of the oral tissue	None	Strong
Texture	Softness	Force required to compress the product with the molars	Hard	Soft
	Overall	An overall assessment expressed by considering all the attributes	Low	High

### Statistical Analysis

Statistical analysis of the results was performed using the STATSOFT 6.0 program (Vigonza, Italy). The significant differences were evaluated by Two-Way analysis of variance (ANOVA), and the mean separation was determined using the Tukey test with respect to time x thesis factor. The sensory data for each attribute were submitted to ANOVA using the software package Statgraphics^®^ *Centurion XVI* (Statpoint Technologies, INC.) using the samples as factors. Significant differences were determined at the alpha level of 0.01 and 0.05 (only for citrus fibers protein and sensory parameters).

## Results and Discussion

### Physicochemical Characterization of the Durum Wheat Whole Flour

Chemical characteristics of whole flour were: moisture 13.15 ± 0.07 g/100 g, ash 1.75 ± 0.10 g/100 g, protein 12.5 ± 0.10 g/100 g. These quality parameters fulfilled the legal requirements (40). Total dietary fiber value was 6.45 ± 0.6 g/100 g, which was constituted by 3.93 ± 0.4 g/100 g of insoluble dietary fiber and 2.52 ± 0.2 g/100 g of soluble dietary fiber. Dry gluten content was 8.4 ± 0.2 g/100 g. Gluten index value was 95.9 ± 5.0 and the value of amylase activity at falling number was low (633 ± 2.0 s). With regard to dry gluten content and relative qualitative index, the whole flour sample exhibited regular gluten quantities but a very high gluten tenacity. As regards color parameters, the values were: brown index (100—L^*^) 15.8 ± 0.2, red index (a^*^) 0.12 ± 0.01, yellow index (b^*^) 18.03 ± 0.04. As for dough rheological characteristics at Mixograph apparatus they were high: mixing time 360 ± 6.0 s and peak dough height 8.0 ± 2.0 M.U. (Mixograph Unit). Consequently, the classification scale (1-8) was good (7 ± 0.0). At Farinograph apparatus the quantity of water absorbed (%) at a 500 B.U. (Brabender Unit) dough consistency (64.6 ± 0.02 %) indicates that the whole flour had a high ability to absorb water due to high fiber content. The values of dough development time 270 ± 6.0 s and dough stability 1,080 ± 11 s were very long due to the good whole flour quality and were in agreement with those reported by other authors (41).

### Characterization of Citrus Fiber

Table 2 reports the chemical characterization of the lemon and blood orange fibers used for the baking process. Both lemon and blood orange had highest levels of total dietary fiber (about 70%), with a balanced content of soluble (about 32%) and insoluble fibers (about 37.5%), differently from data reported for grapefruit, lemon and orange fibers by other authors (12), who reported lower levels of soluble fibers. Citrus fruit are characterized by distinctive concentrations of different minerals, mainly K, Ca, and P (42). Thus, the technological process applied for the preparation of the citrus fibers produced dried flours with 6.63 ± 0.15 g/100 g and 5.67 ± 0.15 g/100 g ash content with a relevant alkalinity index of 55.5 ± 0.08 and 52.7 ± 0.06 for the lemon and blood orange fibers, respectively. The moisture, total protein and sugar values were approximately in lines with values reported by other authors (12, 17).

**Table 2:** Chemical characterization of citrus (lemon and blood orange) fiber used in bread making.

**Parameter**	**Citrus fibers**	
	**Lemon**	**Blood orange**
Total dietary fiber (g/100 g)	70.7 ± 1.0n.s.	70.5 ± 1.0n.s.
Soluble dietary fiber (g/100 g)	31.9 ± 0.05b	33.2 ± 0.05a
Insoluble dietary fiber (g/100 g)	38.8 ± 0.05a	37.3 ± 0.05b
Moisture (g/100 g)	8.84 ± 0.02a	7.85 ± 0.02b
Ash (g/100 g)	6.62 ± 0.15a	5.66 ± 0.15b
Alkalinity of total ash (index)	55.5 ± 0.08a	52.7 ± 0.06b
Protein (g/100 g)	5.69 ± 0.26B	6.48 ± 0.25A
Sugars (g/100 g)	4.21 ± 0.22n.s.	4.01 ± 0.20n.s.
Pectin (g/100 g)^b^	2.15 ± 0.10a	1.60 ± 0.10b
WBC (%)	803 ± 0.00b	804 ± 0.00a

*Data expressed as the mean ± standard deviation. Different letters in the same row are significantly different lower case, p < 0.01; upper case, p< 0.05; n.s., not significant. WBC, water binding capacity*.

### Dietary Fiber Content in Breads

Table 3 reports total, soluble, and insoluble dietary fiber content in bread samples after baking. The highest level of total dietary fiber of citrus fiber (Table 2) had the advantage that small addition to the whole flour did not modify the technological properties of the resulting dough. Furthermore, it resulted in a significant increase in total fiber level in bread. The highest values of total, soluble, and insoluble fiber were recorded in the bread with 2.0% of citrus fibers, even if these thesis did not differ statistically from the others.

**Table 3:** Dietary fiber content after baking in bread samples.

**Sample code**	**Total dietary fiber (g/100 g)**	**Soluble dietary fiber (g/100 g)**	**Insoluble dietary fiber (g/100 g)**
Control	5.93 ± 0.10b	2.32 ± 0.04b	3.61 ± 0.06b
1.5LF	7.10 ± 0.22a	2.86 ± 0.06a	4.24 ± 0.16a
2.0LF	7.41 ± 0.04a	2.99 ± 0.06a	4.42 ± 0.08a
1.5OF	7.01 ± 0.08a	2.87 ± 0.06a	4.14 ± 0.02a
2.0OF	7.33 ± 0.06a	2.96 ± 0.03a	4.37 ± 0.03a
1.0 + 1.0OLF	7.32 ± 0.02a	2.99 ± 0.07a	4.33 ± 0.05a

*1.5LF, 2.0LF sample codes for breads with 1.5 or 2% of lemon fiber, respectively; 1.5OF 2.0OF sample codes for breads with 1.5 or 2% of blood orange fiber, respectively; 1.0 + 1.0OLF sample code for bread with 1 + 1% of lemon and blood orange fiber. Data expressed as the mean ± standard deviation. Different letter in the same column indicates significant difference (p < 0.01)*.

All the theses with citrus fiber exceeded 6.0% of total dietary fiber content, which is the minimum required limit to indicate that a food is “high in fibre,” as required by Regulation (EC) No 1924/2006 of the European Parliament and of the Council on nutrition and health claims made on foods (35).

Therefore, addition of lemon and/or blood orange citrus fibers used in this study as ingredients to produce fiber-enriched bread can be helpful and interesting in integrating the whole flour with valuable and balanced sources of both soluble and insoluble dietary fiber fractions, including pectins.

### Quality Parameters of Breads and the Evolution During Storage

Table 4 reports the *p-*values for all physico-chemical parameters studied on the breads with respect to storage time x thesis factor, while Tables 5, 6 showed the results of the physical and chemical properties of the industrial breads in the MAP conditions during 120 days of storage, respectively. The specific volumes and weights of the loaves were not significant for any of the 3 factors of variability (thesis (A), storage time (B), and their interaction (A × B) (Table 4). No significant differences in the specific volumes and weights were shown among the bread samples and during the storage time, whatever the type and level of citrus fibers, probably due to the use of wheat whole durum flour. These findings are not in agreement with those already reported (12). Wheat white bread is bulkier and less heavy than that made with durum wheat whole semolina, and this fact strongly and negatively impacted the contributions of the orange fiber. The ratio between the height and diameter of the loaves is used in the baking industry to parameterize any failure of the dough (Table 5). The h/d ratio was significant respect to all the 3 factors of variability, even if with different *p* levels (*p* ≤ 0.001 for theses, *p* ≤ 0.01 for storage time and their interaction) (Table 4). During the storage, the control showed the greatest h/d ratio (approximately 3.4) due to the absence of citrus flour that leads to a high dough tenacity. The other bread samples, as expected, exhibited a lower ratio, even if they are not statistically different from the control, with the exception of sample 1 + 1OLF after 30 days of storage (Table 5).

**Table 4:** Analysis of variance of the physico-chemical, color and microbiological parameters studied on the loaves (*p*-values).

**Factors of variability**	**Degrees of freedom**	**Specific volume**	**Specific weight**	**h/d ratio**	**Porosity**	**Internal structure**	**Crust thickness**	**External firmness**	**Moisture**	**pH**	**TA**	**HMF**	**Crust**			**Crumb**			**Yeast and mold**	**Total viable count**
													L^*^	a^*^	b^*^	L^*^	a^*^	b^*^		
Thesis (A)	5	0.123	0.132	0.000	0.000	0.049	0.000	0.000	0.000	0.000	0.000	0.000	0.000	0.085	0.000	0.321	0.000	0.000	0.000	0.000
Storage time (B)	4	0.174	0.139	0.003	0.000	0.003	0.009	0.000	0.000	0.000	0.000	0.000	0.000	0.000	0.000	0.000	0.000	0.000	0.000	0.000
A x B	20	0.834	0.977	0.003	0.000	0.003	0.010	0.000	0.000	0.000	0.000	0.000	0.000	0.035	0.000	0.000	0.005	0.000	0.000	0.000

**Table 5:** Evaluation of properties of bread samples produced using different levels of lemon (LF) and blood orange (OF) fibers, during storage.

**Days of storage**	**Samples**	**Specific volume (cm^**3**^/g)**	**Specific weight (g/cm^**3**^)**	**h/d ratio**	**Porosity (1–8)^**a**^**	**Internal structure (1–2)^**b**^**	**Crust thickness (mm)**	**External firmness (kg/cm^**2**^)**
0	Control	2.48 ± 0.10	0.40 ± 0.02	3.40 ± 0.08ab	7a	1	4.67 ± 0.58abc	2.39 ± 0.52abc
	1.5LF	2.31 ± 0.05	0.43 ± 0.01	3.43 ± 0.09ab	7a	2	4.51 ± 0.02abc	2.36 ± 0.17abcd
	2.0LF	2.60 ± 0.40	0.39 ± 0.05	3.35 ± 0.10abc	6ab	1	5.00 ± 0.01a	2.42 ± 0.31ab
	1.5OF	2.61 ± 0.03	0.38 ± 0.00	3.43 ± 0.02ab	6ab	1	4.95 ± 0.13ab	2.08 ± 0.10abcdefg
	2.0OF	2.59 ± 0.14	0.39 ± 0.02	3.17 ± 0.07abc	6ab	1	4.33 ± 0.59abc	2.27 ± 0.28abcde
	1.0+1.0OLF	2.58 ± 0.52	0.40 ± 0.07	2.93 ± 0.08bc	7a	2	4.34 ± 0.58abc	2.12 ± 0.26abcdefg
30	Control	2.56 ± 0.09	0.39 ± 0.01	3.57 ± 0.11a	7a	1	4.34 ± 0.57abc	1.49 ± 0.16ghij
	1.5LF	2.41 ± 0.08	0.42 ± 0.01	3.10 ± 0.16abc	7a	2	4.17 ± 0.29abc	1.88 ± 0.08abcdefg
	2.0LF	2.43 ± 0.05	0.41 ± 0.01	3.12 ± 0.02abc	6ab	1	5.00 ± 0.03a	1.77 ± 0.26bcdefg
	1.5OF	2.57 ± 0.04	0.39 ± 0.01	3.23 ± 0.25abc	7a	1	4.66 ± 0.56abc	1.69 ± 0.06cdefgh
	2.0OF	2.47 ± 0.16	0.41 ± 0.03	3.12 ± 0.27abc	7a	1	4.34 ± 0.60abc	1.59 ± 0.09efghij
	1.0+1.0OLF	2.33 ± 0.09	0.43 ± 0.02	2.77 ± 0.08c	7a	2	4.01 ± 0.01abc	0.97 ± 0.08j
60	Control	2.72 ± 0.18	0.37 ± 0.02	3.16 ± 0.16abc	7a	1	4.34 ± 0.61abc	1.42 ± 0.11hij
	1.5LF	2.55 ± 0.07	0.39 ± 0.01	3.06 ± 0.20abc	7a	2.	4.01 ± 0.02abc	1.53 ± 0.14fghij
	2.0LF	2.64 ± 0.13	0.38 ± 0.02	3.04 ± 0.21abc	6ab	1	5.00 ± 0.01a	1.62 ± 0.26efghij
	1.5OF	2.65 ± 0.03	0.38 ± 0.00	2.90 ± 0.16bc	7a	1	4.35 ± 0.57abc	1.59 ± 0.05efghij
	2.0OF	2.54 ± 0.12	0.39 ± 0.02	3.10 ± 0.40abc	7a	1	3.68 ± 0.58bc	1.67 ± 0.05defghi
	1.0+1.0OLF	2.55 ± 0.05	0.39 ± 0.01	3.17 ± 0.15abc	7a	2	4.00 ± 0.01abc	1.01 ± 0.17ij
90	Control	2.52 ± 0.05	0.40 ± 0.01	3.43 ± 0.15ab	7a	1	3.99 ± 0.10abc	1.61 ± 0.18efghij
	1.5LF	2.52 ± 0.03	0.40 ± 0.00	2.97 ± 0.20bc	7a	2	4.01 ± 0.02abc	2.54 ± 0.06a
	2.0LF	2.58 ± 0.26	0.39 ± 0.01	2.97 ± 0.12abc	6ab	1	4.67 ± 0.58abc	2.03 ± 0.11abcdefg
	1.5OF	2.68 ± 0.07	0.37 ± 0.01	3.13 ± 0.08abc	7a	1	4.01 ± 0.02abc	1.79 ± 0.15bcdefg
	2.0OF	2.52 ± 0.06	0.40 ± 0.01	2.99 ± 0.11abc	7a	1	3.73 ± 0.31bc	2.24 ± 0.02abcdef
	1.0+1.0OLF	2.50 ± 0.54	0.41 ± 0.01	3.07 ± 0.25abc	7a	2	3.99 ± 0.01abc	2.11 ± 0.05abcdefg
120	Control	2.51 ± 0.01	0.40 ± 0.00	3.37 ± 0.14abc	7a	1	4.01 ± 0.02abc	2.07 ± 0.09abcdefg
	1.5LF	2.53 ± 0.09	0.40 ± 0.01	3.38 ± 0.07ab	7a	2	4.00 ± 0.02abc	1.76 ± 0.30bcdefg
	2.0LF	2.55 ± 0.08	0.40 ± 0.09	3.15 ± 0.15abc	7a	1.	4.42 ± 0.58abc	2.17 ± 0.28abcdef
	1.5OF	2.53 ± 0.04	0.40 ± 0.01	2.99 ± 0.18abc	7a	1	3.95 ± 0.23abc	1.94 ± 0.11abcdefg
	2.0OF	2.54 ± 0.20	0.40 ± 0.03	3.07 ± 0.14abc	7a	1	3.50 ± 0.51c	1.93 ± 0.06abcdefg
	1.0+1.0OLF	2.07 ± 0.60	0.41 ± 0.00	2.92 ± 0.14bc	7a	2	3.84 ± 0.29ab	2.37 ± 0.27abcd

*1.5LF, 2.0LF sample codes for breads with 1.5 or 2% of lemon fiber, respectively; 1.5OF 2.0OF sample codes for breads with 1.5 or 2% of blood orange fiber, respectively; 1.0 + 1.0OLF sample code for bread with 1% +1 % of lemon and blood orange fiber. Data expressed as the mean ± standard deviation. Different letter in the same column indicates significant difference (p < 0.01)*.

a *1, most porous; 8, least porous*.

b *1, regular; 2, irregular*.

**Table 6:** Evaluation of the chemical characteristics of the bread samples produced using different levels of citrus fiber during storage.

**Days of storage**	**Samples**	**Moisture (g/100 g)**	**pH**	**TA^**a**^ (ml NaOH N/10)**	**HMF (mg/kg dry matter)**
0	Control	40.57 ± 0.07ab	5.88 ± 0.06efghij	7.72 ± 0.59abcdefg	65.4 ± 5.09b
	1.5LF	39.38 ± 0.09abc	5.96 ± 0.04abcde	7.42 ± 0.03bcdefghi	85.9 ± 0.56a
	2.0LF	38.53 ± 0.05abcd	5.96 ± 0.04abcde	8.43 ± 0.12ab	65.6 ± 4.49b
	1.5OF	39.43 ± 0.01abc	5.84 ± 0.01ijkl	7.88 ± 0.44abcde	60.0 ± 1.25bc
	2.0OF	40.03 ± 0.14abc	5.90 ± 0.01defghi	8.56 ± 0.33ab	47.4 ± 1.65def
	1.0+1.0OLF	41.58 ± 0.25a	5.85 ± 0.01hijkl	8.78 ± 0.02a	44.8 ± 2.10b
30	Control	39.47 ± 0.09abc	5.93 ± 0.01bcdefg	7.00 ± 0.22cdefghilm	16.9 ± 4.72hil
	1.5LF	38.37 ± 0.07abcd	5.98 ± 0.03abc	7.72 ± 0.02abcdefg	49.9 ± 1.23cdef
	2.0LF	34.19 ± 0.27abcdef	6.00 ± 0.01ab	8.12 ± 0.60abcd	51.0 ± 4.95cde
	1.5OF	32.43 ± 7.14cdefgh	5.93 ± 0.02bcdefg	7.69 ± 0.35abcdefgh	22.9 ± 2.09hi
	2.0OF	39.49 ± 0.12abc	5.90 ± 0.01defghi	8.12 ± 0.13abcd	44.4 ± 0.99ef
	1.0+1.0OLF	37.29 ± 4.24abcde	5.86 ± 0.04fghijk	8.27 ± 0.43abc	39.8 ± 3.38ef
60	Control	30.30 ± 1.54efghi	5.99 ± 0.01abc	6.93 ± 0.01defghilm	9.99 ± 1.17l
	1.5LF	30.30 ± 1.54efghi	5.99 ± 0.01abc	6.05 ± 0.09mn	8.21 ± 1.43l
	2.0LF	27.84 ± 0.26fghij	6.02 ± 0.03a	6.50 ± 0.18ghilmn	13.2 ± 4.28il
	1.5OF	24.80 ± 0.34hijk	5.96 ± 0.01abcd	7.00 ± 0.10cdefghilm	15.6 ± 0.06hil
	2.0OF	19.98 ± 3.57k	5.83 ± 0.03ijkl	7.39 ± 0.10bcdefghil	14.7 ± 2.56hil
	1.0+1.0OLF	22.46 ± 0.76jk	5.95 ± 0.00abcde	6.58 ± 0.36fghilmn	22.7 ± 11.3hi
90	Control	28.81 ± 2.90fghij	5.87 ± 0.01fghijk	5.40 ± 0.09n	10.3 ± 1.04l
	1.5LF	27.91 ± 0.25fghij	5.93 ± 0.02bcdefgh	6.09 ± 0.09lmn	40.3 ± 0.18ef
	2.0LF	23.22 ± 1.83ijk	6.00 ± 0.01abc	5.59 ± 0.29n	37.9 ± 11.9fg
	1.5OF	23.64 ± 0.33ijk	5.86 ± 0.02ghijk	6.39 ± 0.10hilmn	12.6 ± 0.75il
	2.0OF	25.70 ± 1.34ghijk	5.94 ± 0.01bcdef	6.38 ± 0.20ilmn	9.37 ± 1.89l
	1.0+1.0OLF	26.93 ± 5.02fghijk	5.92 ± 0.01cdefgh	6.64 ± 0.29efghilmn	26.1 ± 2.29gh
120	Control	33.16 ± 1.81bcdefg	5.80 ± 0.01kl	8.18 ± 0.01abcd	57.4 ± 2.01bcd
	1.5LF	31.63 ± 0.07defgh	5.78 ± 0.04l	7.95 ± 0.50abcd	42.9 ± 1.57ef
	2.0LF	23.22 ± 1.83ijk	6.00 ± 0.01abc	7.52 ± 0.11abcdefghi	38.6 ± 0.94f
	1.5OF	31.53 ± 0.56defgh	5.80 ± 0.02kl	7.85 ± 0.32abcdef	11.4 ± 0.53il
	2.0OF	32.87 ± 0.67bcdefg	5.81 ± 0.02jkl	8.39 ± 0.12ab	48.5 ± 4.81cdef
	1.0+1.0OLF	32.84 ± 0.10cdefg	5.81 ± 0.01jkl	8.54 ± 0.12ab	17.2 ± 0.84hil

*1.5LF, 2.0LF sample codes for breads with 1.5 or 2% of lemon fiber, respectively; 1.5OF 2.0OF sample codes for breads with 1.5 or 2% of blood orange fiber, respectively; 1.0 + 1.0OLF sample code for bread with 1 +1% of lemon and blood orange fiber. Data expressed as the mean ± standard deviation. Different letter in the same column indicates significant difference (p < 0.01)*.

a *Titratable acidity expressed on dry matter*.

After baking (t0), the 50% of the thesis showed a better developed crumb porosity. Starting from 30 days of storage, the porosity performance of 1.5 and 2.0 OF bread samples decreased (value 7), whereas the 2.0 LF sample maintained good characteristics until 90 days of storage (Table 5).

As regards the bread internal structure was significant both respect to the theses factor (*p* ≤ 0.05) and for the other two factors of variability (*p* ≤ 0.01) (Table 4). Only the samples 1.5 LF and 1.0 + 1.0OLF showed an irregular structure both after baking and during storage. The addition of blood orange fiber decreased the loaf volume but did not deteriorate the crumb characteristics (43) (Table 5).

Regarding the crust thickness, this parameter was significant respect to theses (*p* ≤ 0.001), times (*p* ≤ 0.01) and their interaction (*p* ≤ 0.05) (Table 4). Only the sample 2.0 OF showed a minor crust thickness after 60 days of storage (Table 5).

Concerning to the external firmness it was significant respect to the all factors of variability (Table 4). The storage time significantly affected the external firmness. After baking (t0), all bread samples showed values ranging from 2.08 to 2.42 kg/cm^2^. At 30 days of storage and up to 60 days, a slight reduction was observed in all samples. Then, the values of the external firmness increased in all bread samples. From 30 days after baking and up to 60 days, the moisture in the loaf moved centripetally, causing a softening of the crust and underlying the crumb, producing a decrease in the firmness of the loaves. Then, the moisture balanced again in different parts of the loaves and the texture gradually increased, reaching a high value after 120 days (Table 5).

Table 6 reports the moisture content, pH, TA and HMF content of the bread samples during the entire storage time. The moisture content was significant respect to the all factors of variability (Table 4). The moisture content ranged from approximately 38.5–41.6% at the beginning, but no significant differences were found between the control bread and the other bread samples. At 60 days of storage, the moisture content decreased up to approximately 30% in the control and in the bread with lemon fiber. Bread samples produced with orange fiber and with the mixed fiber highlight the lowest moisture content (about 20%) but only 2.0 OF and 1.0+1.0OLF were significantly different from the control bread. At 90 and 120 days of storage, no remarkable differences were evident between the bread samples, with the exception of 120 days in the 2.0 LF sample, which had the lowest moisture level (Table 6).

The effect of the addition of citrus fiber in bread samples on pH and on titratable acidity seems to be more related to the storage time rather than the recipe. No significant differences in pH were found between the control bread and other bread samples, except for 2.0LF at 90 and 120 days of storage. The pH of this bread sample remains constant throughout the storage time.

Concerning to the TA it was significant at the highest level respect to the all 3 factors of variability (Table 4). The TA ranged from approximately 7.4–8.8 at the beginning. After it decreased gradually up to 90 days of storage while increasing up to the initial levels, afterwards (Table 6).

For the first time the HMF level in durum wheat whole semolina bread was reported. In control bread the HMF was higher (about 65.4 mg/kg dry matter) than those determined in durum wheat bread (39.7 ± 2.22) (31). Differences both in recipe and in chemical characteristics of durum wheat whole semolina are responsible for the different HMF level (44). The HMF content was significant for all 3 factors of variability (Table 4).

The HMF content (Table 5) ranged from approximately 44.8–85.9 mg/kg at the beginning, and significant differences were found between the control bread and the 1.5 LF and 2.0 OF bread. Storage caused significant differences in the HMF levels in all bread samples. HMF decreased with storage up to 60 days, increasing up to 120 days of storage, showing a similar trend respect to TA. This trend, is the result of formation and degradation reactions of HMF (45) influenced by the pH, the temperature and the presence and concentration of sugars and aminoacids (26, 46, 47). The HMF behavior was similar to those observed for TA and the two parameters shows a good correlation (*r*^2^ = 0.5200). It is known that during the Maillard reaction several acids (i.e., formic, acetic) were formed as degradation products (48). Similar result was highlighted during storage of durum wheat bread (31).

Table 7 reports the color parameters of the crust and crumb of bread samples produced using various doses of blood orange and lemon citrus fibers. The crust lightness was significant respect to the all factors of variability (Table 4). At time 0, the 1.0+1.0OLF bread samples had the darkest crust values, whereas the other bread samples had the greatest L^*^ values. At times 30 and 60, the L^*^ crust values were lower for all bread samples. A similar trend was reported for durum wheat bread fortified with pectins and flavonoids (49). At time 90, the crust lightness values remained equal, whereas after 120 days, all of the bread samples showed the greatest L^*^ values.

**Table 7:** Color parameters of breads produced using various doses of blood orange and lemon citrus fibers.

**Days of storage**	**Samples**	**Crust**			**Crumb**		
		L^*^	a^*^	b^*^	L^*^	a^*^	b^*^
0	Control	45.36 ± 3.21abcde	26.44 ± 1.59a	31.40 ± 2.57abcde	78.31 ± 1.96ab	7.25 ± 0.46e	33.04 ± 1.18a
	1.5LF	45.28 ± 0.40bcde	26.06 ± 0.78ab	30.89 ± 1.11abcd	77.77 ± 1.05ab	7.80 ± 0.30cde	33.14 ± 0.56a
	2.0LF	48.85 ± 1.09ab	26.46 ± 0.69a	32.19 ± 1.65abc	78.68 ± 0.22ab	7.91 ± 0.50cde	33.10 ± 1.11a
	1.5OF	47.38 ± 1.49abc	26.37 ± 0.55ab	32.28 ± 1.72abc	77.45 ± 1.63ab	7.59 ± 0.38de	33.01 ± 0.61a
	2.0OF	49.71 ± 0.14a	25.75 ± 0.58ab	32.99 ± 0.34ab	79.78 ± 0.38a	7.24 ± 0.30e	32.74 ± 0.03ab
	1.0+1.0OLF	39.05 ± 0.81ghijkl	25.50 ± 0.67ab	27.93 ± 0.73cde	77.46 ± 0.45ab	8.36 ± 0.26bcde	33.80 ± 0.67a
30	Control	41.65 ± 0.20defg	27.11 ± 0.37a	30.37 ± 0.54abcd	74.39 ± 1.08abcd	8.74 ± 0.15abcd	34.53 ± 0.70a
	1.5LF	45.55 ± 1.53abcd	26.03 ± 0.34ab	31.91 ± 1.11abc	74.45 ± 3.18abcd	9.16 ± 0.14abc	34.84 ± 1.39a
	2.0LF	40.32 ± 1.04fghij	24.78 ± 1.24abc	28.29 ± 1.04bcde	73.66 ± 0.46bcde	8.88 ± 0.16abcd	33.54 ± 0.29a
	1.5OF	40.99 ± 0.56efgh	24.78 ± 1.24abc	34.29 ± 0.47a	74.31 ± 0.91abcd	8.66 ± 0.29abcd	28.29 ± 1.04cde
	2.0OF	41.62 ± 2.00defg	24.67 ± 0.89abc	28.39 ± 1.99bcde	74.34 ± 4.18abcd	8.60 ± 0.23abcde	33.40 ± 0.63a
	1.0+1.0OLF	35.61 ± 0.79klmn	24.46 ± 0.03abc	27.56 ± 0.59cde	75.49 ± 1.02abc	7.96 ± 0.27cde	33.99 ± 0.35a
60	Control	36.09 ± 1.10ijklmn	16.51 ± 0.63h	16.79 ± 0.55j	71.45 ± 1.57cdef	3.58 ± 0.38g	28.43 ± 0.28cde
	1.5LF	33.64 ± 1.19no	17.79 ± 1.44gh	15.90 ± 0.73j	71.28 ± 0.96cdef	4.70 ± 0.40fg	29.51 ± 0.41cd
	2.0LF	40.37 ± 0.98fghi	17.46 ± 0.42gh	18.82 ± 0.68hij	67.65 ± 4.17fgh	4.46 ± 0.63fg	27.05 ± 1.41cdef
	1.5OF	37.82 ± 0.72ghijklmn	18.75 ± 0.85efgh	18.13 ± 0.08hij	67.81 ± 0.46efgh	4.78 ± 0.05fg	27.75 ± 0.57cdef
	2.0OF	34.85 ± 0.28lmno	17.83 ± 1.54gh	17.00 ± 0.33ij	68.10 ± 0.35efgh	4.54 ± 0.76fg	27.26 ± 1.09cdef
	1.0+1.0OLF	30.65 ± 0.79o	17.88 ± 0.46gh	15.08 ± 0.15j	67.57 ± 1.27fgh	4.99 ± 0.30f	27.59 ± 0.57cdef
90	Control	36.07 ± 1.21ijklmn	18.23 ± 1.15fgh	17.57 ± 0.66ij	66.86 ± 0.17fghi	4.37 ± 0.29fg	27.08 ± 0.43cdef
	1.5LF	38.12 ± 0.48ghijklm	18.70 ± 0.29efgh	18.56 ± 0.68hij	66.68 ± 0.87fghij	5.26 ± 0.29f	27.41 ± 0.32cdef
	2.0LF	33.54 ± 0.27no	17.87 ± 1.06gh	16.19 ± 0.65j	66.16 ± 0.70fghij	5.16 ± 0.46f	27.21 ± 0.78cdef
	1.5OF	36.98 ± 1.14hijklmn	17.48 ± 0.13gh	17.57 ± 0.19ij	62.80 ± 1.20ghij	4.90 ± 0.25fg	26.08 ± 0.33ef
	2.0OF	34.13 ± 2.46mno	16.76 ± 0.72h	15.88 ± 0.67j	61.25 ± 1.32ij	5.02 ± 0.15f	24.83 ± 0.32f
	1.0+1.0OLF	35.97 ± 0.68jklmn	18.73 ± 0.42efgh	17.55 ± 0.58ij	68.26 ± 0.50efgh	5.05 ± 0.46f	28.09 ± 0.48cde
120	Control	39.52 ± 1.08ghijk	23.17 ± 1.10bcd	26.71 ± 2.43defg	61.59 ± 2.70ij	8.79 ± 0.59abcd	27.30 ± 2.23cdef
	1.5LF	45.70 ± 0.21abcd	21.90 ± 1.01cde	26.94 ± 2.37def	64.08 ± 1.76ghij	9.78 ± 0.04a	29.90 ± 0.85bc
	2.0LF	35.24 ± 1.33klmn	21.73 ± 1.11cde	22.62 ± 2.91fgh	62.36 ± 1.37hij	9.11 ± 0.22abc	26.32 ± 0.64ef
	1.5OF	43.95 ± 1.52cdef	21.11 ± 2.37def	26.49 ± 2.23defg	66.54 ± 0.90fghij	9.78 ± 0.80a	29.12 ± 1.03cde
	2.0OF	35.91 ± 0.60klmn	21.79 ± 0.17cde	23.82 ± 1.03efg	68.49 ± 0.46defg	9.14 ± 0.31abc	29.78 ± 0.23bc
	1.0+1.0OLF	37.56 ± 0.22ghijklmn	20.50 ± 1.73defg	21.81 ± 1.95ghi	60.86 ± 0.48j	9.39 ± 0.09ab	26.67 ± 0.95def

*1.5LF, 2.0LF sample codes for breads with 1.5 or 2% of lemon fiber, respectively; 1.5OF 2.0OF sample codes for breads with 1.5 or 2% of blood orange fiber, respectively; 1.0 + 1.0OLF sample code for bread with 1 +1% of lemon and blood orange fiber. Data expressed as the mean ± standard deviation. Different letters in the same column indicates significant difference (p < 0.01)*.

The crust redness it was significant for storage time and the interaction between theses and storage time, instead crust yellowness was significant respect to all the factors of variability (Table 4).

No significant differences were found in the a^*^ and b^*^ parameters after baking or at time 30 between the crust color of the control and other bread samples. Therefore, low values were shown in the 1.0+1.0OLF crust samples. At times 60 and 90, the a^*^ and b^*^ parameters demonstrated a lowering trend for all of the bread samples. After 120 days of storage, an increase in the a^*^ and b^*^ values was observed, with intermediate values between 0 and 30, 60 and 90 days.

The crumb lightness was not significant respect to one factor of variability (theses), but was significant for storage time and their interaction (Table 4). The effect of the addition of citrus fibers on the L^*^ parameter of crumbs after baking and after 30 days of storage were not significant. At time 60, the L^*^ value was reduced. The control and 1.5LF bread sample had the greatest values.

The a^*^ and b^*^ crumb color parameters were significant respect to the all factors of variability (Table 4). After time 60 the a^*^ values decreased and then increased again to 120 days of storage (Table 7).

As browning index parameter has been considered 100–L^*^ (22). In this study, no linear correlation was found between HMF and 100–L^*^ (*r*^2^ = 0.348) according to results reported for durum wheat bread (31), but in contrast with those reported for dough baked at various times even if product with the same colors can have different HMF levels (23).

### Microbiological Assay

Table 8 reports microbial dynamics of the different groups throughout the storage period. The values of CO_2_ and O_2_ in the ATM packaged samples remained unchanged for the entire storage period (data not shown). The yeast, mold and total viable count were significant respect to all the factors of variability (Table 4).

**Table 8:** Mean log_10_ of microbial population counts of breads produced using various doses and types of citrus fibres during storage.

**Days of storage**	**Samples**	**Yeast and mold**	**Total viable count**
0	Control	1.08 ± 0.06ij	0.96 ± 0.01k
	1.5LF	1.14 ± 0.13ij	1.08 ± 0.40k
	2.0LF	1.11 ± 0.15ij	1.08 ± 0.26k
	1.5OF	1.02 ± 0.15i	2.00 ± 0.15i
	2.0OF	1.22 ± 0.26ij	1.78 ± 0.28ij
	1.0+1.0OLF	1.27 ± 0.09hij	1.29 ± 0.06jk
30	Control	1.72 ± 0.23ghij	2.05 ± 0.08i
	1.5LF	1.87 ± 0.14fghi	2.07 ± 0.02i
	2.0LF	2.07 ± 0.05efgh	2.31 ± 0.04hi
	1.5OF	1.72 ± 0.08ghij	2.69 ± 0.08gh
	2.0OF	2.36 ± 0.04defg	3.42 ± 0.17def
	1.0+1.0OLF	2.53 ± 0.15cdefg	2.64 ± 0.07gh
60	Control	2.09 ± 0.19efg	2.86 ± 0.11fgh
	1.5LF	2.59 ± 0.06cdef	2.80 ± 0.23gh
	2.0LF	2.74 ± 0.11bcde	3.06 ± 0.13efg
	1.5OF	2.78 ± 0.10bcde	3.08 ± 0.10efg
	2.0OF	2.86 ± 0.13bcde	3.86 ± 0.15cd
	1.0+1.0OLF	3.07 ± 0.11abcd	2.75 ± 0.30gh
90	Control	2.15 ± 0.02efg	3.12 ± 0.18efg
	1.5LF	3.11 ± 0.17abcd	3.83 ± 0.21cd
	2.0LF	3.30 ± 0.08abc	3.71 ± 0.21cd
	1.5OF	3.14 ± 0.22abcd	3.72 ± 0.22cd
	2.0OF	3.16 ± 0.21abcd	4.15 ± 0.14abc
	1.0+1.0OLF	3.33 ± 0.12abc	3.88 ± 0.21bcd
120	Control	2.47 ± 0.16defg	3.61 ± 0.37cde
	1.5LF	3.43 ± 0.31ab	4.12 ± 0.23abc
	2.0LF	3.69 ± 0.49a	4.12 ± 0.18abc
	1.5OF	3.33 ± 0.05abc	3.99 ± 0.05abcd
	2.0OF	3.49 ± 0.18ab	4.51 ± 0.23a
	1.0+1.0OLF	3.79 ± 0.28a	4.46 ± 0.14ab

*1.5LF, 2.0LF sample codes for breads with 1.5 or 2% of lemon fiber, respectively; 1.5OF 2.0OF sample codes for breads with 1.5 or 2% of blood orange fiber, respectively; 1.0 + 1.0OLF sample code for bread with 1 + 1% of lemon and blood orange fiber.Data expressed as the mean of microbial log counts (log_10_ cfu/g) ± standard deviation. Different letters in the same column indicates significant difference (p < 0.01)*.

No mold growth was visible in well-sealed packages. At the end of storage, the greatest total viable count (TVC) and yeast counts were achieved. The yeast and mold counts exhibited values of approximately 1 log_10_ cfu/g for all of the bread samples prior to 30 days of storage. From 30 through 120 days of storage, a gradual increase in the yeast and mold counts was detected in all samples, achieving the greatest values of 3.79 log_10_ cfu/g for sample 1.0+1.0 OLF, whereas the control sample had the lowest at 2.47 log_10_ cfu/g. A similar trend was reported for TVC, with values of 4.51 and 4.46 log_10_cfu/g for samples 2.0 OF and 1.0+1.0 OLF, respectively. In the control bread, the yeast, mold and TVC remained slightly lower during all storage times possibly due to the absence of fiber, although the differences are not significant up to 60 days of storage.

Trials on freshly baked bread enriched with potato flour with 10 days of storage showed similar trends, with mold values of approximately 1–2 log_10_ cfu/g and bacteria of approximately 5 log_10_ cfu/g for bread enriched with 10% Irish potato flour (50).

Aerobic plate counts (log_10_ cfu/g) of bread enriched with plantain flour after baking ranged from 3.20 to 4.50 (51). These counts were minimal and would not constitute health hazards, but are greater compared with the data obtained in this study. This is the first time that microbiological data on industrial bread enriched with fiber have been reported after a long storage period. Confirming the importance of packaging conditions and good practices during industrial production to prevent contamination and microbial proliferation after processing.

### Sensory Evaluation

For the first time the sensory profiles of a durum wheat whole semolina bread and durum wheat whole semolina bread with citrus fiber were determined.

The sensory profile both of control and of the fortified bread with citrus fiber was peculiar due to the ingredients used, such as durum wheat whole semolina and citrus fiber. A previous study (52) reported that durum wheat breads, have peculiar sensory features, different from those of common wheat breads (38).

Table 9 reports the mean scores of all attributes for t0, while for the other days of storage only the statistically significant attributes. From the ANOVA results of the sensory data, the freshly baked samples were significant different for the attributes humidity, bitter, overall and citrus aroma and flavor.

**Table 9:** Mean scores of the sensory attributes at the different days of storage.

**Days of storage**	**Attributes^**a**^**	**Control**	**1.5OF**	**2.0OF**	**1.5LF**	**2.0LF**	**1.0+1.0OLF**
0	Crumb color	6.58 ± 1.73	6.33 ± 1.61	7.00 ± 1.81	6.33 ± 1.61	6.41 ± 1.51	7.16 ± 1.59
	Alveolar structure	5.25 ± 2.22	5.16 ± 1.70	4.41 ± 1.44	5.25 ± 2.30	5.75 ± 1.60	5.00 ± 2.09
	Humidity	6.58 ± 1.44b	6.16 ± 1.80ab	6.08 ± 2.11ab	6.00 ± 1.48ab	4.91 ± 2.27a	5.83 ± 2.41ab
	Bread aroma	5.83 ± 1.85	5.58 ± 2.07	5.58 ± 1.73	6.25 ± 1.54	5.58 ± 1.88	6.00 ± 1.86
	Yeasty aroma	4.41 ± 2.19	4.41 ± 2.11	3.91 ± 2.23	4.83 ± 1.75	5.16 ± 1.64	5.50 ± 1.68
	Citrus aroma	1.00 ± 0.00a	3.83 ± 2.37c	3.66 ± 2.15c	2.08 ± 0.90ab	2.83 ± 1.47bc	2.16 ± 1.34ab
	Off-odor	2.50 ± 1.62	3.16 ± 2.76	3.25 ± 1.82	3.00 ± 1.60	2.50 ± 1.57	2.91 ± 1.83
	Sweet	3.75 ± 2.18	2.58 ± 1.56	3.25 ± 1.82	3.50 ± 1.73	3.75 ± 2.26	3.50 ± 1.45
	Salty	4.16 ± 2.25	4.33 ± 2.27	4.33 ± 1.37	4.91 ± 1.38	4.58 ± 2.47	5.16 ± 1.70
	Sour	3.41 ± 2.31	4.25 ± 2.09	4.08 ± 2.23	3.00 ± 1.71	3.66 ± 2.64	3.66 ± 2.31
	Bitter	2.91 ± 1.51a	3.58 ± 2.15ab	5.33 ± 2.46b	3.58 ± 2.23ab	3.75 ± 2.53ab	3.91 ± 2.07ab
	Astringent	4.66 ± 2.15	4.25 ± 2.73	4.16 ± 2.41	4.16 ± 2.55	4.50 ± 2.20	4.41 ± 2.71
	Softness	5.66 ± 1.61	4.58 ± 2.15	4.58 ± 2.71	5.16 ± 1.95	4.08 ± 2.11	5.08 ± 2.11
	Bread flavor	5.58 ± 2.23	4.33 ± 2.06	4.50 ± 1.62	4.66 ± 2.31	4.91 ± 2.15	4.83 ± 2.33
	Yeasty flavor	3.66 ± 1.87	3.75 ± 1.48	3.25 ± 1.48	4.08 ± 2.64	3.91 ± 1.68	4.16 ± 1.59
	Citrus flavor	1.00 ± 0.00a	3.50 ± 2.50b	2.83 ± 2.50b	2.83 ± 2.15b	3.25 ± 2.00b	2.91 ± 2.01b
	Off-flavor	2.41 ± 2.34	1.91 ± 2.92	2.33 ± 1.81	3.33 ± 2.26	3.08 ± 2.84	2.50 ± 2.67
	Overall	4.08 ± 1.81ab	5.25 ± 1.78b	4.00 ± 2.19a	3.83 ± 1.71a	3.91 ± 1.70a	4.00 ± 2.18a
30	Crumb color	6.16 ± 1.64ab	5.25 ± 1.82a	7.00 ± 1.76b	6.50 ± 1.51ab	7.16 ± 1.75b	6.75 ± 1.42b
	Humidity	3.83 ± 1.85ab	4.75 ± 1.66b	3.41 ± 1.16ab	3.75 ± 1.96ab	3.75 ± 1.66ab	3.25 ± 1.96a
	Citrus aroma	1.00 ± 0.00a	3.08 ± 1.98b	2.91 ± 2.02b	2.75 ± 1.86b	3.00 ± 1.86b	2.41 ± 1.62b
	Softness	3.25 ± 1.14a	4.33 ± 1.30b	2.33 ± 0.98a	3.25 ± 1.48a	3.00 ± 1.28a	2.66 ± 1.07a
	Citrus flavor	1.00 ± 0.00a	3.50 ± 2.15b	2.83 ± 1.95b	2.83 ± 1.99b	3.25 ± 2.09b	2.91 ± 1.68b
	Overall	4.08 ± 1.73ab	5.25 ± 1.60b	4.00 ± 1.13a	3.83 ± 1.59a	3.91 ± 1.51a	4.00 ± 1.48a
60	Humidity	3.83 ± 1.47b	4.00 ± 1.76b	3.58 ± 1.31ab	2.33 ± 0.98a	2.91 ± 1.44ab	3.00 ± 2.04ab
	Citrus aroma	1.00 ± 0.00a	3.58 ± 2.23bcd	4.00 ± 2.04d	2.33 ± 1.23b	2.66 ± 1.15bc	3.66 ± 1.97cd
	Sweet	4.00 ± 1.65ab	3.08 ± 1.38ab	4.25 ± 1.14b	4.00 ± 1.81ab	3.08 ± 1.00ab	3.00 ± 1.65a
	Softness	3.58 ± 1.56b	3.58 ± 1.51b	3.33 ± 1.56ab	2.16 ± 1.19a	2.75 ± 1.14ab	3.33 ± 2.06ab
	Bread flavor	5.00 ± 1.71b	3.75 ± 1.82ab	4.08 ± 1.73ab	3.75 ± 1.29ab	2.83 ± 1.11a	3.66 ± 1.61a
	Citrus flavor	1.00 ± 0.00a	4.75 ± 2.45b	4.58 ± 2.35b	3.58 ± 2.07b	4.08 ± 1.73b	5.00 ± 2.30b
	Overall	4.91 ± 1.56b	4.08 ± 1.38ab	3.91 ± 1.38a	3.41 ± 1.24a	3.00 ± 1.28a	3.91 ± 1.88ab
90	Crumb color	5.08 ± 1.00ab	4.58 ± 0.79a	5.83 ± 0.58bc	5.41 ± 1.31bc	5.25 ± 1.06abc	5.91 ± 0.79c
	Alveolar structure	3.83 ± 1.27b	4.16 ± 1.34b	4.08 ± 1.68b	2.58 ± 1.16a	3.66 ± 1.23ab	3.33 ± 1.44ab
	Humidity	2.58 ± 1.16a	3.75 ± 0.62b	3.25 ± 1.06ab	2.66 ± 1.30a	3.25 ± 0.97ab	2.75 ± 1.22a
	Citrus aroma	1.00 ± 0.00a	2.33 ± 1.07bc	3.00 ± 1.48c	2.00 ± 0.95b	2.41 ± 1.08bc	2.58 ± 1.38bc
	Sour	2.58 ± 1.31abc	3.00 ± 1.13bc	1.83 ± 0.83a	3.25 ± 1.29c	2.33 ± 0.89bc	2.50 ± 1.00abc
	Astringent	3.41 ± 1.44b	3.33 ± 1.07b	2.16 ± 1.19a	3.08 ± 1.31ab	2.83 ± 1.40ab	3.25 ± 1.54ab
	Yeasty flavor	2.58 ± 0.79a	4.00 ± 1.76b	2.58 ± 1.00a	3.00 ± 0.85a	3.00 ± 1.04a	2.50 ± 1.00a
	Citrus flavor	1.00 ± 0.00a	2.91 ± 0.90b	4.16 ± 1.59c	3.08 ± 1.88b	3.25 ± 1.54bc	2.83 ± 1.03b
	Overall	3.00 ± 0.74a	3.16 ± 0.94a	4.66 ± 1.37b	3.91 ± 1.38ab	3.75 ± 1.06ab	3.33 ± 1.15a
120	Citrus aroma	1.00 ± 0.00a	2.50 ± 1.45b	3.16 ± 1.95b	2.41 ± 1.62b	2.25 ± 1.48b	2.58 ± 1.51b
	Yeasty flavor	4.25 ± 1.96b	3.25 ± 1.54ab	3.50 ± 1.88ab	3.50 ± 1.78ab	2.83 ± 1.11a	3.91 ± 1.78ab
	Citrus flavor	1.00 ± 0.00a	2.91 ± 1.83b	3.25 ± 1.91bc	2.75 ± 1.60b	4.50 ± 2.47c	2.91 ± 2.02b
	Overall	3.58 ± 1.51ab	4.50 ± 1.51b	3.50 ± 1.17ab	3.16 ± 0.72a	3.75 ± 1.54ab	3.33 ± 1.50a

*1.5LF, 2.0LF sample codes for breads with 1.5 or 2% of lemon fiber, respectively; 1.5OF 2.0OF sample codes for breads with 1.5 or 2% of blood orange fiber, respectively; 1.0 + 1.0OLF sample code for bread with 1 + 1% of lemon and blood orange fiber*.

a *For t0 all attributes are shown, for the other days of storage only the statistically significant attributes*.

*Data expressed as the mean ± standard deviation. Values marked with different letters in the same row are significant difference (p ≤ 0.05)*.

Panelist of bread samples containing up to 30% of fine bran, with a fiber level ranging from 1.5 to 3% did not find significant differences in crumb color, texture and flavor when compared with the control (whole wheat flour) (53). Results reported in this study showed that bread samples containing up to about 7.3% of total dietary fiber, due to fortification with citrus fiber, did not show changes in crumb color, alveolar structure, softness and bread flavor, suggesting citrus fiber as a good source of dietary fiber that did not significantly change the main sensory attributes of durum wheat whole semolina bread.

The samples 1.5LF and 1+1OLF had the low intensity of citrus aroma; the sample 2.0OF showed the high intensity of bitter while the addition up to 1.5% of orange fiber improves the overall intensity scores.

At 30 days of storage, the samples were significant different for the attributes crumb color, humidity, softness, overall, citrus aroma, and flavor. Samples with citrus fiber had the same intensity of citrus aroma and flavor. The bread sample 1.5OF had the highest intensity of softness due to its highest humidity intensity score.

At 60 days, the bread samples were significant different for the attributes humidity, sweet, softness, overall, bread flavor, citrus aroma, and flavor. Samples 1.5 LF and 2.0LF showed the high intensity of citrus aroma, while the sample 1.5OF and control had the highest intensity of softness strictly related to their highest humidity intensity score. Moreover, the control bread showed the highest intensity of bread flavor and overall.

At 90 days of storage, the sensory profile of samples changes. In fact, on the nine sensory attributes, four attributes, such as alveolar structure, sour, astringent and yeasty flavor, for the first time were statistically significant. Other significant different attributes were crumb color, humidity, citrus aroma and flavor and overall. The samples with orange fiber had an alveolar structure similar with control bread. Also the astringent intensity was similar in control and 1.5OF bread samples. Bread sample 1.5OF had the highest intensity of yeasty flavor. The overall scores of bread samples were similar.

At 120 days, the samples were significant different only for the attributes citrus aroma and flavor, yeasty flavor and overall, suggesting a leveling of the samples' sensory profile. The bread sample with the highest addition of lemon fiber (2.0LF) showed the highest intensity of citrus flavor and the lowest of yeasty flavor, while the sample 1.5OF had the highest intensity of overall. For the attribute citrus aroma all the bread samples fortified with citrus fiber showed the same intensity. The control sample had the highest intensity of yeasty flavor. The attribute of yeasty flavor differentiated the samples after 90 days of storage. This is probably due to bread aging, ascribed to a reduction of the intensity of citrus aroma and flavor. These changes were more evident in control bread produced without citrus fiber. Moreover, it is important to highlight that bread samples, during storage, did not develop off-odor and off-flavor, and up to 30 days of storage overall scores of bread samples were similar independently to recipe.

## Conclusions

For the first time in this paper, was studied the possibility to add citrus fiber to produce a wheat whole durum bread fortified in dietary fiber. The addition of citrus fiber up to 2% allowed to produce, generally, wheat whole durum bread with quality parameters, similar to control bread. No deep differences were highlighted between bread samples. Long storage time in MAP conditions significantly affected the bread characteristics independently from the addition of citrus fiber and allowed to maintain the microbiological safety of the bread samples.

This study showed that the addition of citrus industry by-product fibers in durum wheat whole semolina is a possible strategy and a good prospect to produce “high fibre” wholemeal durum wheat bread to increase the nutritional value and the dietary fiber intake. These results, moreover, should be interesting for the possibility to produce a “high in fibre” durum wheat bread with a long shelf-life.

Finally, the use of citrus fibers in bread making can be considered as an environmental-friendly alternative for the reuse and valorisation of citrus processing wastes and by-products.

## Author Contributions

AS and EA planned the experiments, surveyed laboratories, interpreted and discussed the results, and co-wrote the manuscript. SM performed laboratory flour analysis, acquired bread data during storage, performed the microbiological assay, and the statistical analyses. AM performed sensorial analysis, interpreted, and discussed sensory results. SB performed laboratory chemical analysis and acquired bread data during storage. SF performed laboratory citrus fiber analysis and participated in the critical revision of manuscript. BF and PR conceived the idea, designed the study and participated in the critical revision of manuscript.

### Conflict of Interest Statement

The authors declare that the research was conducted in the absence of any commercial or financial relationships that could be construed as a potential conflict of interest.

## References

[1] Jacobs DR Jr Meyer KA Kushi LH Folsom AR. Whole-grain intake may reduce the risk of ischemic heart disease death in postmenopausal women. The Iowa Women's Health Study. Am J Clin Nutr. (1998) 68:248–57. 9701180 10.1093/ajcn/68.2.248

[2] Montonen J Knekt P Jarvinen R Aromaa A Reunanen A. Whole-grain and fiber intake and the incidence of type 2 diabetes. Am J Clin Nutr. (2003) 77:622–29. 10.1093/ajcn/77.3.622 12600852

[3] Jacobs DR Jr Andersen LF Blomhoff R. Whole-grain consumption is associated with a reduced risk of noncardiovascular, noncancer death attributed to inflammatory diseases in the Iowa Women's Health Study. Am J Clin Nutr. (2007) 85:1606–14. 10.1093/ajcn/85.6.1606 17556700

[4] Kendall CWC Esfahani A Jenkins DJA The link between dietary fibre and human health. Food Hydrocoll. (2010) 24:42–8. 10.1016/j.foodhyd.2009.08.002

[5] Slavin JL. Dietary fiber and body weight. Nutrition (2005) 21:3. 10.1016/j.nut.2004.08.018 15797686

[6] Bozzetto L Costabile G Della Pepa G Ciciola P Vetrani C Vitale M. Dietary fibre as a unifying remedy for the whole spectrum of obesity-associated cardiovascular risk. Nutrients (2018) 10:7. 10.3390/nu10070943 30037123 PMC6073249

[7] Gòmez M Ronda F Blanco CA Caballero PA Apesteguia A Effect of dietary fibre on dough rheology and bread quality. Eur Food Res Technol. (2003) 216:51–6. 10.1007/s00217-002-0632-9

[8] Grigelmo-Miguel N Carreras-Boladeras E Martin-Belloso O Influence of the addition of peach dietary fiber in composition, physical properties and acceptability of reduced-fat muffins. Food Sci Technol Int. (2001) 7:425–31. 10.1106/FLLH-K91M-1G34-Y0EL

[9] Vitali D Dragojević IV Šebečić B Effects of incorporation of integral raw materials and dietary fibre on the selected nutritional and functional properties of biscuits. Food Chem. (2009) 114:4 10.1016/j.foodchem.2008.11.032

[10] Ajila CM Leelavathi K Rao U Improvement of dietary fiber content and antioxidant properties in soft dough biscuits with the incorporation of mango peel powder. J Cereal Sci. (2008) 48:319–26. 10.1016/j.jcs.2007.10.001

[11] Stojceska V Ainsworth P Plunkett A İbanoğlu E İbanoğlu Ş Cauliflower by-products as a new source of dietary fibre, antioxidants and proteins in cereal based ready-to-eat expanded snacks. J Food Eng. (2008) 87:554–63. 10.1016/j.jfoodeng.2008.01.009

[12] Figuerola F Hurtado ML Estevez AM Chiffelle I Asenjo F Fibre concentrates from apple pomace and citrus peel as potential fibre sources for food enrichment. Food Chem. (2005) 91:395–401. 10.1016/j.foodchem.2004.04.036

[13] Angioloni A Collar C Physicochemical and nutritional properties of reduced-caloric density high-fibre breads. LWT - Food Sci Technol. (2011) 44:747–58. 10.1016/j.lwt.2010.09.008

[14] Nassar AG Abd El-Hamied AA El-Naggar EA Effect of citrus by-products flour incorporation on chemical, rheological and organoleptic characteristics of biscuits. WRJAS (2008) 4:612–16.

[15] Larrea MA Chang YK Martinez-Bustos F Some functional properties of extruded orange pulp and its effect on the quality of cookies. LWT - Food Sci Technol. (2005) 38: 213–20. 10.1016/j.lwt.2004.05.014

[16] Bilgicli N Ibanoglu S Herken EN Effect of dietary fibre addition on the selected nutritional properties of cookies. J Food Eng. (2007) 78:86–9. 10.1016/j.jfoodeng.2005.09.009

[17] Romero-Lopez MR Osorio-Diaz P Bello-Perez LA Tovar J Bernardino-Nicanor A Fiber concentrate from mexican orange (*Citrus sinensis* L.) bagase: characterization and application as bakery product ingredient. Int J Mol Sci. (2011) 12: 2174–86. 10.3390/ijms12042174 21731434 PMC3127110

[18] Rada-Mendoza M Sanz ML Olano A Villamiel M Study on nonenzymatic browning in cookies, crackers and breakfast cereals by maltulose and furosine determination. Food Chem. (2004) 85:605–09. 10.1016/j.foodchem.2003.07.002

[19] Arena E Fallico B Maccarone E Thermal damage in blood orange juice: kinetics of 5-hydroxymethyl-2-furancarboxaldehyde formation. Int J Food Sci Technol. (2001) 36:145–51. 10.1046/j.1365-2621.2001.00436.x

[20] Fallico B Zappalà M Arena E Verzera A Effect of conditioning on HMF content in unifloral honeys. Food Chem. (2004) 85:305–13. 10.1016/j.foodchem.2003.07.010

[21] Ramirez-Jiménez A Guerra-Hernàndez E Garcìa-Villanova B. (2000). Browning indicators in bread. J Agric Food Chem. (2000) 48:4176–81. 10.1021/jf9907687 10995333

[22] Resmini P Pellegrino L Pagani MA De Noni I Formation of 2-acetyl-3-d-glucopyranosylfuran (glucosylisomaltol) from nonenzymatic browning in pasta drying. Ital J Food Sci. (1993) 5:341–53.

[23] Ramírez-Jiménez A García-Villanova B Guerra-Hernández E Hydroxymethylfurfural andmethylfurfural content of selected bakery products. Food Res Int. (2000) 33:833–38. 10.1016/S0963-9969(00)00102-2

[24] Abraham K Gürtler R Berg K Heinemeyer G Lampen A Appel KE. Toxicology and risk assessment of 5-Hydroxymethylfurfural in food. Mol Nutr Food Res. (2011) 55:667–78. 10.1002/mnfr.201000564 21462333

[25] Arena E Brighina S Mazzaglia A Spina A Muccilli S Giannone V Use of a natural low Na salt in durum wheat bread. Ital J Food Sci. (2015) 77–81.

[26] Ameur LA Mathieu O Lalanne V Trystram G Birlouez-Aragon I Comparison of the effects of sucrose and hexose on furfural formation and browning in cookies baked at different temperatures. Food Chem. (2007) 101:1407–16. 10.1016/j.foodchem.2006.03.049

[27] Gökmen V Açar ÖC Köksel H Açar J Effects of dough formula and baking conditions on acrylamide and hydroxymethylfurfural formation in cookies. Food Chem. (2007) 104:1136–42. 10.1016/j.foodchem.2007.01.008

[28] Gökmen V Açar ÖC Serpen A Morales FJ Effect of leavening agents and sugars on the formation of hydroxymethylfurfural in cookies during baking. Eur Food Res Technol. (2008) 226:1031–37. 10.1007/s00217-007-0628-6

[29] Association of Official Analytical Chemists Method 991.43 for soluble and insoluble dietary fibre. (2000).

[30] Sgrulletta D De Stefanis E Simultaneous evaluation of quality parameters of durum wheat (*Triticum durum*) by near infrared reflectance spectroscopy. Ital J Food Sci. (1997) 4:295–301.

[31] Spina A Brighina S Muccilli S Mazzaglia A Rapisarda P Fallico B Arena E Partial replacement of NaCl in bread from durum wheat (*Triticum turgidum* L. subsp. durum Desf.) with KCl and yeast extract: evaluation of quality parameters during long storage. Food Bioprocess Tech. (2015) 8:1089–101. 10.1007/s11947-015-1476-1

[32] Caggia C Lanza CM Bellomo SE Pannuzzo P Lo Bianco M Restuccia C Blood Orange slices packaged with films of different permeabilities: chemical, microbiological and sensory studies. Ital J Food Sci. (2004) 16:275–92.

[33] JECFA Joint (FAO/WHO) Expert Committee on Food Additives, 2007 Compendium of Food Additive Specifications. FAO JECFA Monographs 4. Food and Agriculture Organization of the United Nations, Rome. Available online at: http://www.fao.org/docrep/010/a1447e/a1447e00.htm. (Accessed July 20, 2017)

[34] Medcalf DG Gilles KA Wheat starches I. Comparison of physicochemical properties. AACC Cereal Chem. (1965) 42:558–68.

[35] Regulation (EC) No 1924/2006 of the European Parliament and of the Council of 20 December 2006 on nutrition and health claims made on foods.

[36] Lefebvre D Gabriel V Vayssier Y Fontagne'-Faucher C Simultaneous HPLC determination of sugars, organic acids and ethanol in sourdough process. LWT Food Sci Tech. (2002) 35: 407–14. 10.1006/fstl.2001.0859

[37] Lanza CM Mazzaglia A Scacco A Pecorino B Changes in sensory and instrumental features of industrial Sicilian bread during storage. Ital J Food Sci. (2011) 23:6–12.

[38] Giannone V Giarnetti M Spina A Todaro A Pecorino B Summo C. Physico-chemical properties and sensory profile of durum wheat Dittaino PDO (Protected Designation of Origin) bread and quality of re-milled semolina used for its production. Food Chem. (2018) 241:242–49. 10.1016/j.foodchem.2017.08.096 28958525

[39] Raffo A Pasqualone A Sinesio F Paoletti F Quaglia G Simeone R Influence of durum wheat cultivar on the sensory profile and staling rate of Altamura bread. Eur Food Res Technol. (2003) 218:49–55. 10.1007/s00217-003-0793-1

[40] Italian Presidential Decree No 187/2001 Decreto del 9 febbraio 2001, n. 187. Regolamento per la Revisione della Normativa Sulla Produzione e Commercializzazione di Sfarinati e Paste Alimentari, a Norma Dell'articolo 50 Della Legge 22 Febbraio 1994, n. 146. Official Journal of Italian Republic, 117, 6–12 (in Italian).

[41] Pasqualone A Laddomada B Centomani I Paradiso VM Minervini D Caponio F Bread making aptitude of mixtures of re-milled semolina and selected durum wheat milling by-products. LWT Food Sci Technol. (2017) 78:151–9. 10.1016/j.lwt.2016.12.032

[42] Fabroni S Rapisarda P Gli agrumi. scchi. In: Tribulato E Inglese P editors. Milan:Bayer CropScience S.r.l. (2012). p. 438–51.

[43] Miller RA Increased yield of bread containing citrus peel fiber. Cereal Chem. (2011) 88:174–78. 10.1094/CCHEM-11-10-0161

[44] Rufián-Henares JA Delgado-Andrade C Morales FJ Assessing the Maillard reaction development during the toasting process of common flours employed by the cereal products industry. Food Chem. (2009) 114:93–9. 10.1016/j.foodchem.2008.09.021

[45] Fallico B Arena E Zappalà M Prediction of honey shelf life. J Food Quality (2009) 32:352–68. 10.1111/j.1745-4557.2009.00253.x

[46] Belitz HB Grosch W Schieberle P Food Chemistry. 3rd ed Berlin: Springer (2004). p. 258–82.

[47] Hamzaloğlu A Gökmen V Investigation and kinetic evaluation of the reactions of hydroxymethylfurfural with amino and thiol groups of amino acids. Food Chem. (2018) 240: 354–60. 10.1016/j.foodchem.2017.07.131 28946283

[48] Van Boekel MAJS Brands CMJ Heating of Sugar-casein Solutions: Isomerization and Maillard Reactions. In: O'Brien J Nursten HE Crabbe MJC Ames JM editors. The Maillard Reaction in Foods and Medicine. Cambridge: Woodhead Publishing (2005). p. 154–8.

[49] Spina A Muccilli S Arena E Brighina S Fallico B Giannone V Durum wheat breads enriched with citrus fruits pectin and flavonoids. Ital J Food Sci. (2015) 72-6.

[50] Ijah UJJ Auta HS Aduloju MO Aransiola SA. Microbiological, nutritional, and sensory quality of bread produced from wheat and potato flour blends. Int J Food Sci. (2014) 2014:671701. 10.1155/2014/671701 26904642 PMC4745549

[51] Olaoye OA Onilude AA Idowu OA Quality characteristics of bread produced from composite flours of wheat, plantain and soybeans. Afr J Biotechnol. (2006) 5:1102–6.

[52] Pasqualone A Italian durum wheat breads. In: Clerici MTPS editor. Bread Consumption and Health. Hauppage, NY: Nova Science Publisher Inc (2012). p. 57–79.

[53] Al-Saqer JM Sidhu JS Al-Hooti SN Instrumental texture and baking quality of high-fiber toast bread as affected by added wheat mill fractions. J Food Process Preserv. (2000) 24:1–16. 10.1111/j.1745-4549.2000.tb00402.x

